# The potential distribution of *Bacillus anthracis* suitability across Uganda using INLA

**DOI:** 10.1038/s41598-022-24281-8

**Published:** 2022-11-19

**Authors:** V. A. Ndolo, D. Redding, M. A. Deka, J. S. Salzer, A. R. Vieira, H. Onyuth, M. Ocaido, R. Tweyongyere, R. Azuba, F. Monje, A. R. Ario, S. Kabwama, E. Kisaakye, L. Bulage, B. Kwesiga, V. Ntono, J. Harris, J. L. N. Wood, A. J. K. Conlan

**Affiliations:** 1 Disease Dynamics Unit, Department of Veterinary Medicine, University of Cambridge, Madingley Rd, Cambridge, Cambridgeshire UK; 2 Centre for Biodiversity and Environment Research, Department of Genetics, Evolution and Environment, University College London, London, UK; 3 College of Veterinary Medicine Animal Resources and Biosecurity, Makerere University, Kampala, Uganda; 4 Uganda National Institute of Public Health, Ministry of Health, Kampala, Uganda; 5 US Centers for Disease Control and Prevention, 1600 Clifton Rd. NE, Atlanta, GA USA

**Keywords:** Ecological epidemiology, Ecological modelling, Microbial ecology

## Abstract

To reduce the veterinary, public health, environmental, and economic burden associated with anthrax outbreaks, it is vital to identify the spatial distribution of areas suitable for *Bacillus anthracis*, the causative agent of the disease. Bayesian approaches have previously been applied to estimate uncertainty around detected areas of *B. anthracis* suitability. However, conventional simulation-based techniques are often computationally demanding. To solve this computational problem, we use Integrated Nested Laplace Approximation (INLA) which can adjust for spatially structured random effects, to predict the suitability of *B. anthracis* across Uganda. We apply a Generalized Additive Model (GAM) within the INLA Bayesian framework to quantify the relationships between *B. anthracis* occurrence and the environment. We consolidate a national database of wildlife, livestock, and human anthrax case records across Uganda built across multiple sectors bridging human and animal partners using a One Health approach. The INLA framework successfully identified known areas of species suitability in Uganda, as well as suggested unknown hotspots across Northern, Eastern, and Central Uganda, which have not been previously identified by other niche models. The major risk factors for *B. anthracis* suitability were proximity to water bodies (0–0.3 km), increasing soil calcium (between 10 and 25 cmolc/kg), and elevation of 140–190 m. The sensitivity of the final model against the withheld evaluation dataset was 90% (181 out of 202 = 89.6%; rounded up to 90%). The prediction maps generated using this model can guide future anthrax prevention and surveillance plans by the relevant stakeholders in Uganda.

## Introduction

Anthrax is a zoonotic disease caused by *Bacillus anthracis*, a Gram-positive, soil-borne, and spore-forming bacterium. In some environmental conditions, *B. anthracis* spores can survive in the soil for years or even decades^1,2^, making long-term control of the disease very difficult. The disease primarily affects both domestic and wild animals^3,4^, mostly herbivores, and can be transmitted to humans when they touch, ingest, or inhale bacterial spores from infected animals, carcasses, or animal by-products^5^. Anthrax is estimated to cause about 20,000–100,000 human infections annually across the world^6^. Approximately 1.1 billion livestock live in areas predicted to be at risk for anthrax globally^6^.

Outbreaks can reduce the economic productivity of the agricultural sector, including dairy production, harm biodiversity, and wildlife conservation efforts, and threaten the food security for communities that consume meat^7^. Effective control of anthrax disease in livestock needs robust surveillance efforts, annual livestock vaccination, and timely outbreak response involving ring vaccination, safe disposal of carcasses, and awareness creation campaigns^3^. There is currently no policy in Uganda concerning the routine annual livestock vaccination against anthrax since the benefits of vaccination against the disease are considered a “private good,” so farmers have to buy vaccines and prophylactic antibiotics privately for their animals, usually during ongoing anthrax outbreaks^3^.

The use of Ecological Niche Model (ENMs) in the fields of conservation biology, epidemiology, and environmental health has increased in recent years. ENMs seek to estimate the association between species presence or absence with environmental variables to predict the probability of the species suitability in non-sampled locations or time periods^8^. ENMs have been used in the past to identify “hotspots” or priority areas likely to be inhabited by disease-causing micro-organisms or vectors^9–11^. It is vital to identify these areas correctly to institute appropriate interventions. Several methods have been created over the past years to build ENMs for *B. anthracis* suitability; these include regression models such as Generalized Linear Models (GLM), Climate Envelope Models (e.g., BIOCLIM), Maximum Entropy Models (e.g., MAXENT), Artificial Neural networks such as SPECIES, and Classification and Regression Trees (e.g., BIOMOD)^12–16^. However, ecological datasets are complex, often requiring sophisticated statistical techniques to analyse them^17^. It is now standard practice to require explicit spatial dependence structures when developing models for complex and non-linear associations between the species and environmental variables and to estimate the sources of uncertainty due to the sampling methods, reporting biases, input data, and other analytical errors^17^. Conventional approaches are limited in their ability to accommodate complex hierarchical dependency structures needed to adjust for spatial autocorrelation within ecological data^18^.

Hierarchical Bayesian models can account for spatial autocorrelation and include spatial random effects that might capture spatial distributions^19^. Posterior predictive distributions obtained from Bayesian models usually require numerical techniques to approach them. Simulation-based methods such as Markov Chain Monte Carlo (MCMC) may be used, but are computationally intensive^20,21^ while the novel Integrated-Nested Laplace Approximation (INLA) approach^22^ offers a better alternative. INLA has gained popularity as a framework for modelling spatial and temporal data of disease-causing organisms (e.g., Rift-Valley Fever and Lassa Fever)^23,24^, as it can produce improved predictions compared to the conventional ENM approaches^18^. However, INLA appears yet to be used to model the spatial distribution of *Bacillus anthracis*.

This study aims to predict the geographical distribution of the species suitability of *B. anthracis* across Uganda using wildlife, livestock, and human data collected from 2004 to 2018. We use a Generalized Additive Model (GAM) using the INLA Bayesian framework to quantify the relationships between the occurrence of *B. anthracis* infection and the environment. Our dataset consolidates a national database of anthrax case records across Uganda built across multiple sectors bridging human and animal partners using a One Health approach.

## Methods

### Study setting

Uganda covers 241,037 km^2^ (4° N, 2° S, 29° E, 35° E) and averages 1100 m above sea level^25^. Uganda has a total population of 34.6 million people, with 7.4 and 27.2 million living in urban and rural areas, respectively^26^. It borders Lake Victoria and has an equatorial climate. The study area is mainly plateau, with a few mountains. About 20 percent of Uganda’s area is covered by swamps and water bodies, including the four Great Lakes of East Africa (Lake Edward, Lake Victoria, Lake Albert, and Lake Kyoga). The country has ten national parks housing a high diversity of wildlife and endangered species^26^. By 2009, agriculture ranked as the second leading contributor to the country’s Gross Domestic Product^27^.

### Surveillance data of *Bacillus anthracis* infection

**Figure 1:**
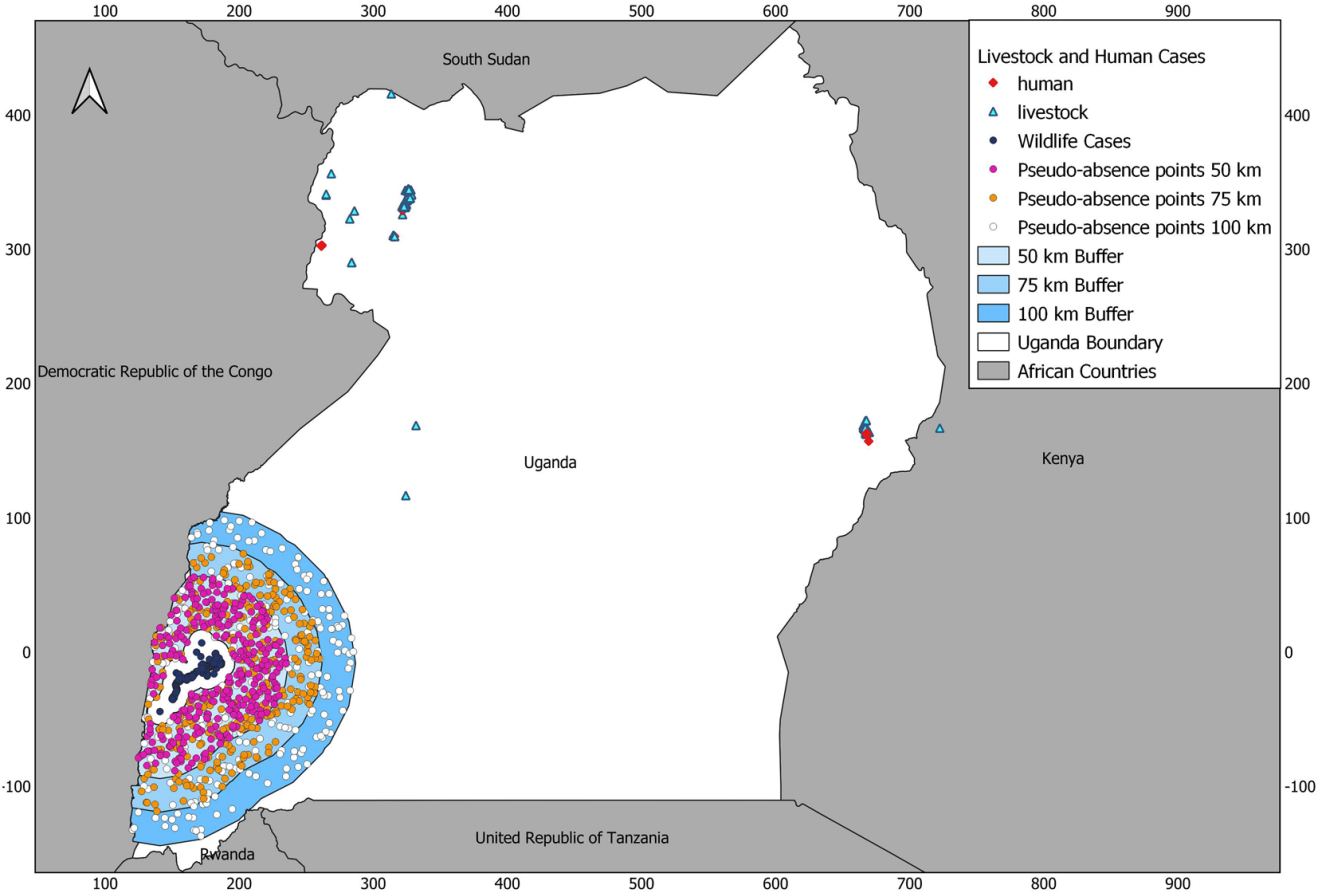
Distribution of anthrax presence and pseudo-absence locations across Uganda. The navy-blue circles show wildlife cases (n = 294) used for model training, the blue triangles show livestock cases (n = 171), and the red diamonds represent human cases (n = 32). The blue polygons show the locations of the 50 km, 75 km, and 100 km buffers which were constructed around the wildlife cases with a distance of 10 km between the buffers and the presence locations. The pink dots show the pseudo-absence points selected within the 50 km buffer, the orange dots show the pseudo-absence points selected within the 75 km buffer, and the white dots show the pseudo-absence points selected within the 100 km buffer. Prediction maps were developed using the Quantum Geographic Information System software (QGIS). URL: https://qgis.osgeo.org (2020).

Surveillance data of livestock and human cases from 2018 were provided by the Ministry of Health (MOH) in Uganda through the Field Epidemiology and Laboratory Training Program and the Uganda Ministry of Agriculture, Animal Industry and Fisheries (MAAIF). Geographical coordinates of anthrax cases among wildlife (from 2004 to 2010) in Queen Elizabeth National Park were obtained from a recently published study^4^. These cases were mapped to show the distribution of anthrax across Uganda (Fig. 1). It is only recently that Uganda has mandated systematic anthrax surveillance across the country following the outbreak that started in 2018. GPS coordinates gathered by field personnel during outbreak responses were used to map outbreak locations. The human anthrax cases were defined based on the CDC’s clinical criteria (signs and symptoms), presumptive laboratory diagnosis (Gram staining), and confirmatory laboratory diagnosis (bacterial culture, immunohistochemistry, ELISA, and PCR)^28^. The animal anthrax cases were also defined based on the clinical presentation, presumptive laboratory diagnosis, and confirmatory laboratory diagnosis. All cases were classified as either ‘probable’ or ‘confirmed,’ with probable defined as cases that met both the clinical and presumptive laboratory diagnostic criteria and confirmed defined as cases that met the clinical and confirmatory laboratory diagnostic criteria. A total of 497 livestock (n = 171), humans (n = 32), and wildlife cases (n = 294), both confirmed (n = 32) and probable (n = 465), occurring from 2004 to 2018 were compiled. All methods were performed in accordance with the relevant guidelines and regulations.

### Environmental variable processing

**Figure 2:**
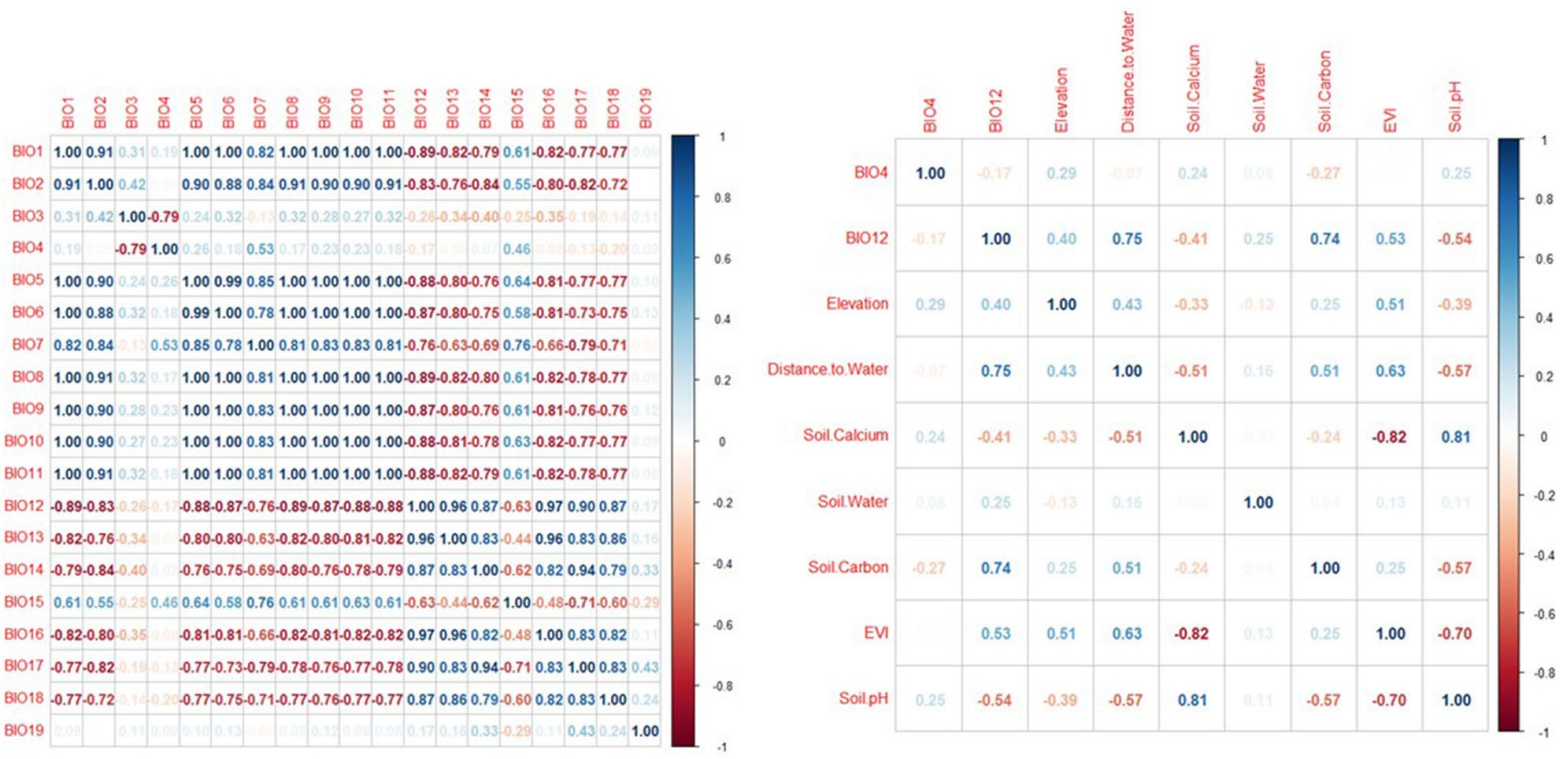
Results of correlation between covariates using Pearson’s correlation test. Correlation between covariates was shown by red numbers (negative correlation) and blue numbers (positive correlation). BIO1 = Annual Mean Temperature, BIO2 = Mean Diurnal Range, BIO3 = Isothermality, BIO4 = Temperature Seasonality, BIO5 = Max Temperature of Warmest Month, BIO6 = Min Temperature of Coldest Month, BIO7 = Temperature Annual Range, BIO8 = Mean Temperature of Wettest Quarter, BIO9 = Mean Temperature of Driest Quarter, BIO10 = Mean Temperature of Warmest Quarter, BIO11 = Mean Temperature of Coldest Quarter, BIO12 = Annual Precipitation, BIO13 = Precipitation of Wettest Month, BIO14 = Precipitation of Driest Month, BIO15 = Precipitation Seasonality, BIO16 = Precipitation of Wettest Quarter, BIO17 = Precipitation of Driest Quarter, BIO18 = Precipitation of Warmest Quarter, BIO19 = Precipitation of Coldest Quarter.

Correlative studies of environmental risk factors for anthrax outbreaks suggest that temperature^6,29–37^, precipitation^6,29–39^, elevation^6,29,31,32,34,35,37–39^, soil (type, calcium concentration, pH, carbon content, and moisture)^6,30,31,33,34,36,37,39–43^, vegetation^6,29,31,34,36–40,42,43^, and hydrology^37,44^ are some of the major drivers of *B. anthracis* suitability. A total of 26 environmental predictors (Fig. 2) were selected for this study based on these known variables. These comprised 19 bioclimatic variables (the mean for the years 1970–2000) collected from the WorldClim database version 2 (https://www.worldclim.org/data/worldclim21.html) at a resolution of 30 s (~ 1 km^2^)^45^. Four soil variables, including exchangeable calcium at a depth of 0–20 cm, soil water availability, soil pH (10×) in H_2_O at a depth of 0 cm, and soil organic carbon at a depth of 0–5 cm, were retrieved from the International Soil Reference and Information Centre (ISRIC) data hub at a resolution of 250 m (https://data.isric.org/geonetwork/srv/eng/catalog.search#/home). Distance to permanent water bodies was derived from a global hydrology map provided by ArcGIS online version 10.6.1^46^. Elevation data of 1 km^2^ in resolution was obtained from the Global Multi-resolution Terrain Elevation Data (GMTED2010) dataset available from the United States Geological Service. Finally, the monthly Enhanced Vegetation Index (EVI) data for the years 2004, 2005, and 2010 (36 tiles in total) were obtained from The Aqua Moderate Resolution Imaging Spectroradiometer (MODIS) Vegetation Indices (MYD13A3 v.6) at a spatial resolution of 1 km (https://lpdaac.usgs.gov/products/myd13a3v006/). The single variable, mean EVI, was calculated in QGIS by averaging all 36 tiles. The EVI minimizes variations in the canopy background and maintains precision over conditions with dense vegetation.

All environmental variables were resampled using the R package ‘Resample’^47^ to a resolution of 1 km and clipped to the extent of Uganda. Since data sampling for wildlife data (used in model training) was not done systematically across the study area, target backgrounds buffers (polygon buffers created at certain radii from the training points and used for the random selection of pseudo-absences) were created for model calibration to reduce sampling bias. As there was no information on the sampling radius, a sensitivity analysis was conducted by creating target backgrounds using circular buffers of radius 50 km, 75 km, and 100 km around the presence points used for model training, leaving 10 km between the presence points and the various buffers (Fig. 1). Quantum Global Information System (QGIS) version 3.16 (https://qgis.org) software was then used to add 294 random pseudo-absence points within each buffer polygon giving a ratio of 1:1 for the training presences to pseudo-absences. A recent study explored how four approaches of pseudo-absence creation affect the performance of models across different species and three model types by building both terrestrial and marine models using boosted regression trees, generalised additive mixed models, and generalised linear mixed models^48^. They then tested four methods for generating pseudoabsences across all the different model types: (1) correlated random walks (RW); (2) reverse correlated RW; (3) sampling pseudoabsences within a buffer area surrounding the presence points; (4) background sampling^48^. The findings of the study suggested that the separation or distance in the environmental space between the presence locations and the pseudoabsences was the most significant driver of the model predictive ability and explanatory power, and thus finding was consistent across the three model types (boosted regression trees, generalised additive mixed models, and generalised linear mixed models) and both the terrestrial and marine habitats^48^.

The values of the environmental variables were then extracted for each presence and pseudo-absence location using the raster package in R. With these, we did an initial data exploration to check for outliers within the covariates, collinearity, and to explore the relationships between the covariates and the response variables (presence or absence of anthrax). Cleveland dot plots were used to check for possible outliers. Following the outlier checks, variance inflation factors (VIF), pairwise plots, and Pearson correlation coefficients with correction for multiple comparisons were used to measure the statistically significant correlation between the covariates (Fig. 2). For variables that were highly correlated (correlation greater than 0.6) or those with high variance inflation (VIF > 3), only one was used in the modelling process. Five variables were selected following this analysis: Temperature seasonality (BIO4), elevation, distance to water, soil calcium, and soil water (Table 1).

**Table 1:** Summary of the environmental variables used.

Variable name	Units	Spatial resolution	Source	Year
BIO1 = Annual Mean Temperature,	°C	30 arc-s	WorldClim database version 2 (https://www.worldclim.org/data/worldclim21.html) (36)	The average for 1970–2000
BIO2 = Mean Diurnal Range,				
BIO3 = Isothermality,				
BIO4 = Temperature Seasonality				
BIO5 = Max Temperature of Warmest Month				
BIO6 = Min Temperature of Coldest Month				
BIO7 = Temperature Annual Range				
BIO8 = Mean Temperature of Wettest Quarter				
BIO9 = Mean Temperature of Driest Quarter				
BIO10 = Mean Temperature of Warmest Quarter				
BIO11 = Mean Temperature of Coldest Quarter				
BIO12 = Annual Precipitation	ml			
BIO13 = Precipitation of Wettest Month				
BIO14 = Precipitation of Driest Month				
BIO15 = Precipitation Seasonality				
BIO16 = Precipitation of Wettest Quarter				
BIO17 = Precipitation of Driest Quarter				
BIO18 = Precipitation of Warmest Quarter				
BIO19 = Precipitation of Coldest Quarter				
Elevation (m)	m	1 km	Global Multi-resolution Terrain Elevation Data (GMTED2010) dataset available from the United States Geological Service	2010
Distance to permanent water bodies (km)	km	1 km	Derived from a global hydrology map provided by ArcGIS online version 10.6.1	2019
Mean Enhanced Vegetation index (EVI)	Units	1 km	The Aqua Moderate Resolution Imaging Spectroradiometer (MODIS) Vegetation Indices (MYD13A3 v.6) (https://lpdaac.usgs.gov/products/myd13a3v006/)	Average of 36 tiles from the years 2004, 2005, and 2010
Soil exchangeable calcium (depth of 0–20 cm)	cmolc/kg	250 m	International Soil Reference and Information Centre (ISRIC) data hub (https://data.isric.org/geonetwork/srv/eng/catalog.search#/home)	
Soil water availability	v%			
Soil pH (× 10) in H2O (depth of 0 cm)	Units (1–14)			
Soil organic carbon (depth of 0–5 cm)	Dg/kg			

### Modelling anthrax suitability across Uganda

QGIS v.3.16 (https://qgis.org) and the R statistical package version 4.1.0^49^ were used to conduct data visualization, cleaning, and model analysis (R code used available in a Github repository: https://github.com/valentinandolo/Uganda-Spatial_Model/tree/master). The wildlife cases (n = 294) were used for model training and testing. The remaining human (n = 32) and livestock (n = 171) cases were used for model evaluation. Since the wildlife case locations were recorded from 2004 to 2010, while the livestock and human cases were recorded in 2018, the latter locations were both spatially and temporally distinct from the wildlife cases, making an excellent basis for block cross-validation of the final model performance^50^. Random partitioning of the data into training and testing sets can inflate the performance of a model and underestimate the error in the spatial prediction evaluation^50^. Block cross-validation uses spatial blocks that separate the testing and training datasets; thus, the method has been suggested to be a good technique for error estimation and a robust approach for measuring a model’s predictive performance^50^.

The INLA package in R was applied to model the suitability of *B. anthracis* across Uganda. INLA calculates the spatial interaction effects using a Stochastic Partial Differential Equation (SPDE) method, which estimates a continuous Gaussian Markov Random Field (GMRF) where the correlation between two locations in space is specified by the Matérn correlation which is explained in more detail elsewhere^51^. The initial step in fitting an SPDE model is the creation of a Constrained Refined Delaunay Triangulation or a mesh to illustrate the spatial process^51^. R-INLA uses a function called ‘inla.mesh.2d()’ that applies a variety of arguments to build the mesh, these include: *loc, loc.domain, boundary, max.edge*, and *cutoff*^51^. The *loc* argument contains the point locations which provide information about the area of study and are used to create the triangulation nodes. Alternatively, a polygon of the study area can be used to identify the extent of the domain via *loc.domain*. We applied the point locations using the *loc* argument. We then specified the boundary of the mesh as a convex hull. We used the *max.edge* argument to specify the maximum edge length for the inner mesh domain/triangles and the outer triangles. We did this by first studying the distribution of distances between the point locations for the training presences and pseudo-absences. Most points were within a distance of about 90–100 km away from each other, thus, a possible guess for the range at which spatial autocorrelation persists was 100 km. We used a distance of 20 km as a range guess to create a finer mesh which has been shown to produce more precise models. We specified the maximum edge length for the inner triangles as 20 km divided by 5 (4 km) and the maximum edge length for the outer triangles as 20 km. Finally, the *cutoff* argument sets the minimum distance allowed between point locations. We divided the maximum edge length for the inner triangles by 5 (4 km divided by 5 = 0.8 km), meaning that points that were closer in distance than 0.8 km were replaced by one vertex to avoid the occurrence of small triangles.

The spatial effect, which is a numeric vector, then links each observation within the data to a spatial location, thus, accounting for region-specific variation that cannot be accounted for by the covariates. Following the recommendation by Lindgren and Rue^52^, multivariate Gaussian distributions with means of zero and a spatially defined covariance matrix were used to model the spatial effect. Several versions of Bayesian hierarchical additive models were created by estimating a Bernoulli generalized additive model (GAM) with and without spatially correlated random effects. The Bernoulli GAM is defined as shown in Eqs. (1) and (2)

1 $${C}_{i} \sim Bernoulli \left({p}_{i}\right),$$

2 $$logit \left({p}_{i}\right)= \alpha +\sum_{j=1}^{m}{\beta }_{j}\left({X}_{j,i}\right)+ \sum_{k=1}^{l}{f}_{k}\left({X}_{k,i}\right)+ {\mu }_{i},$$

where ${C}_{i}$ denotes the observed value, such that: *B. anthracis* presence or absence at a given location *i* (*i* = 1, …, n; n = 588) is given as ${C}_{i}$, where ${C}_{i}$ =1 if *B. anthracis* was present, and ${C}_{i}$=0 if absent. Logit is the link function for binomial family, ${p}_{i}$ is the expected value of the response variable (the probability of *B. anthracis* suitability) at location *i*, α is the intercept, ${X}_{j,i}$ and ${X}_{k,i}$ are the j th and the k th covariates at a location *i*, ${\beta }_{j}$ are the beta coefficients, ${f}_{k}$ are the smooth functions (cubic regression splines) for k th covariates, and ${\mu }_{i}$ is the spatial random effect at the location *i*^53^. The number of variables in linear term (*m*) and the non-liner term (*l*) are different because the variables employed in each term are different. We estimated both linear and non-linear effects for the covariates. Our overall database had 294 *B. anthracis* pseudo-absences generated randomly across the target background buffers to match the number of species presences recorded. Because we had no prior information, a Gaussian prior distribution with a mean of zero (default no effect prior unless data is informative) was applied for all the model parameters. The posterior mean, standard deviation, and 95% credible intervals were estimated for all the parameters.

### Model selection

Several different candidate models were examined. First, a baseline model was built using only the intercept. A second baseline model was then built, which included the intercept and spatial random effects only. Covariates were added to the second baseline model (intercept plus spatial effects model) without any smoothing function (i.e., only linear effects). The contribution of the spatial random effect was then re-examined by taking it out from the model. Smoothing functions were then added to all covariates, and the same procedure was repeated. Model selection was done using this forward stepwise approach. The final model was run using the three different target background buffers to identify the buffer distance with the lowest Deviance Information Criterion (DIC). The various options were assessed using the DIC^54^, Watanabe-Akaike information criterion (WAIC), and the Conditional Predictive Ordinate (CPO). The DIC and WAIC were chosen because they are commonly used to assess model performance by measuring the compromise between the goodness of fit and complexity. For CPO, the logarithmic score (− mean(log (CPO)) (LCPO) was calculated and used^55^. CPO can also be used to conduct internal cross-validation of models using a leave-one-out approach to evaluate the predictive performance of the model. Lower LCPO, DIC, and WAIC estimates suggest superior model performance. Thus, the favoured model had the lowest values across the 3 metrics.

### Model validation and evaluation

Model validation for the favoured model was conducted using an independent evaluation dataset comprising of livestock and human outbreaks occurring at different spatial locations and 8 years after the training data. The omission rate, which indicates the proportion of positive test locations that end up in pixels predicted to be unsuitable for *B. anthracis*^56^, was used to validate the model. A low omission rate indicates good model performance. The threshold for suitability was the probability threshold that maximized the sensitivity and specificity of the model. The sensitivity was then derived by calculating the proportion of positive test locations that end up in pixels predicted to be suitable for *B. anthracis*^56^*.* A high sensitivity indicates good model performance.

### Model prediction

The favoured model selected using the criteria described above was used to generate countrywide prediction maps showing the posterior mean values, standard deviation, and the 95% credible intervals of the probability of *B. anthracis* suitability. The raster package in R was used to make the prediction maps. Bayesian kriging was done by treating all model parameters as random variables to include uncertainty in the prediction^57^. This kriging is built into the INLA framework via the SPDE, which allows a Delaunay triangulation to be constructed around the presence and absence locations within the sampling frame^52^. INLA then conducts model inference and prediction at the same time by considering the prediction points as locations missing the response variable (set to NA)^51^. Following successful model prediction, additional linear interpolation functions then generate the output for the whole study area scaled from 0 to 1.

### Ethical approval

Ethical approval for this study was provided by the Human Biology Research Ethics Committee, University of Cambridge, UK (Ref: HBREC.2019.02) the School of Veterinary Medicine and Animal Resources Institutional Animal Care and Use Committee, Makerere University, Uganda (Ref: SVAREC/21/2019); and the Uganda National Council for Science and Technology. Informed consent was obtained from all subjects and/or their legal guardian(s). All methods were performed in accordance with the relevant guidelines and regulations.

## Results

A total of 497 anthrax cases comprising livestock (n = 171), humans (n = 32), and wildlife cases (n = 294), occurring from 2004 to 2018 were compiled (Fig. 1). Of these, 32 cases were classified as confirmed and 465 cases were probable. The wildlife cases were used to train and calibrate the model. Table 2 shows the results of the candidate models created across the different target background buffers. The 75 km background buffer radius gave the best results in terms of lower DIC values, so it was selected as the final background extent for model calibration. The baseline prediction model that incorporated the spatial random effect had a much lower DIC than the baseline model without it (Table 2) and addition of linear covariates reduced it further. The chosen best model (option 17 in Table 2) had the lowest DIC, WAIC and LCPO compared to the rest of the models. This final model applied a non-linear effect on elevation and linear effects on distance to water, soil calcium, and soil water (Table 2). The spatial random effect was not included in the model.

**Table 2:** Model comparison of the fitted INLA models using linear and non-linear associations across three background buffer radii. The criteria for comparison are *DIC* Deviance Information Criterion, *WAIC* Watanabe-Akaike Information Criterion, *LCPO* Conditional Predictive Ordinate, Variables acronyms are *Distw* Distance to water, *Ca* Soil calcium, *Elev* Elevation, *Swater* Soil water, *BIO4* Temperature Seasonality, $\mu$ Spatial effect.

Buffer radius	Model option	Type	Variables	DIC	WAIC	CPO
50 km	1	Linear	Baseline	817	817	0.69
	2	Linear	Baseline + μ	187	181	0.15
	3	Linear	BIO4 + Distw + Elev + Ca + Swater + μ	85	85	0.07
	4	Linear	BIO4 + Distw + Elev + Ca + Swater	83	84	0.07
	5	Linear	Distw + Elev + Ca + Swater	81	81	0.07
	6	Non-linear	f(Distw) + f(Elev) + f(Ca) + f(Swater)	78	118	1.75
	7	Non-linear	f(Distw) + f(Elev) + f(Ca) + f(Swater) + μ	120	263	5.29
	8	Non-linear	Distw + f(Elev) + Ca + Swater	60	61	0.05
	9	Non-linear	Distw + f(Elev) + Ca + Swater + μ	63	64	0.06
75 km	10	Linear	Baseline	817	817	0.69
	11	Linear	Baseline + μ	187	182	0.15
	12	Linear	BIO4 + Distw + Elev + Ca + Swater + μ	60	75	1.93
	13	Linear	BIO4 + Distw + Elev + Ca + Swater	50	60	0.08
	14	Linear	Distw + Elev + Ca + Swater	49	53	0.07
	15	Non-linear	f(Distw) + f(Elev) + f(Ca) + f(Swater)	108	301	2.20
	16	Non-linear	f(Distw) + f(Elev) + f(Ca) + f(Swater) + μ	184	604	9.79
	17	Non-linear	Distw + f(Elev) + Ca + Swater	44	47	0.05
	18	Non-linear	Distw + f(Elev) + Ca + Swater + μ	53	63	2.95
100 km	19	Linear	Baseline	817	817	0.69
	20	Linear	Baseline + μ	176	170	0.14
	21	Linear	BIO4 + Distw + Elev + Ca + Swater + μ	95	96	0.08
	22	Linear	BIO4 + Distw + Elev + Ca + Swater	95	96	0.08
	23	Linear	Distw + Elev + Ca + Swater	92	92	0.08
	24	Non-linear	f(Distw) + f(Elev) + f(Ca) + f(Swater)	77	161	5.16
	25	Non-linear	f(Distw) + f(Elev) + f(Ca) + f(Swater) + μ	145	613	9.50
	26	Non-linear	Distw + f(Elev) + Ca + Swater	85	85	0.07
	27	Non-linear	Distw + f(Elev) + Ca + Swater + μ	86	86	0.08

**Figure 3:**
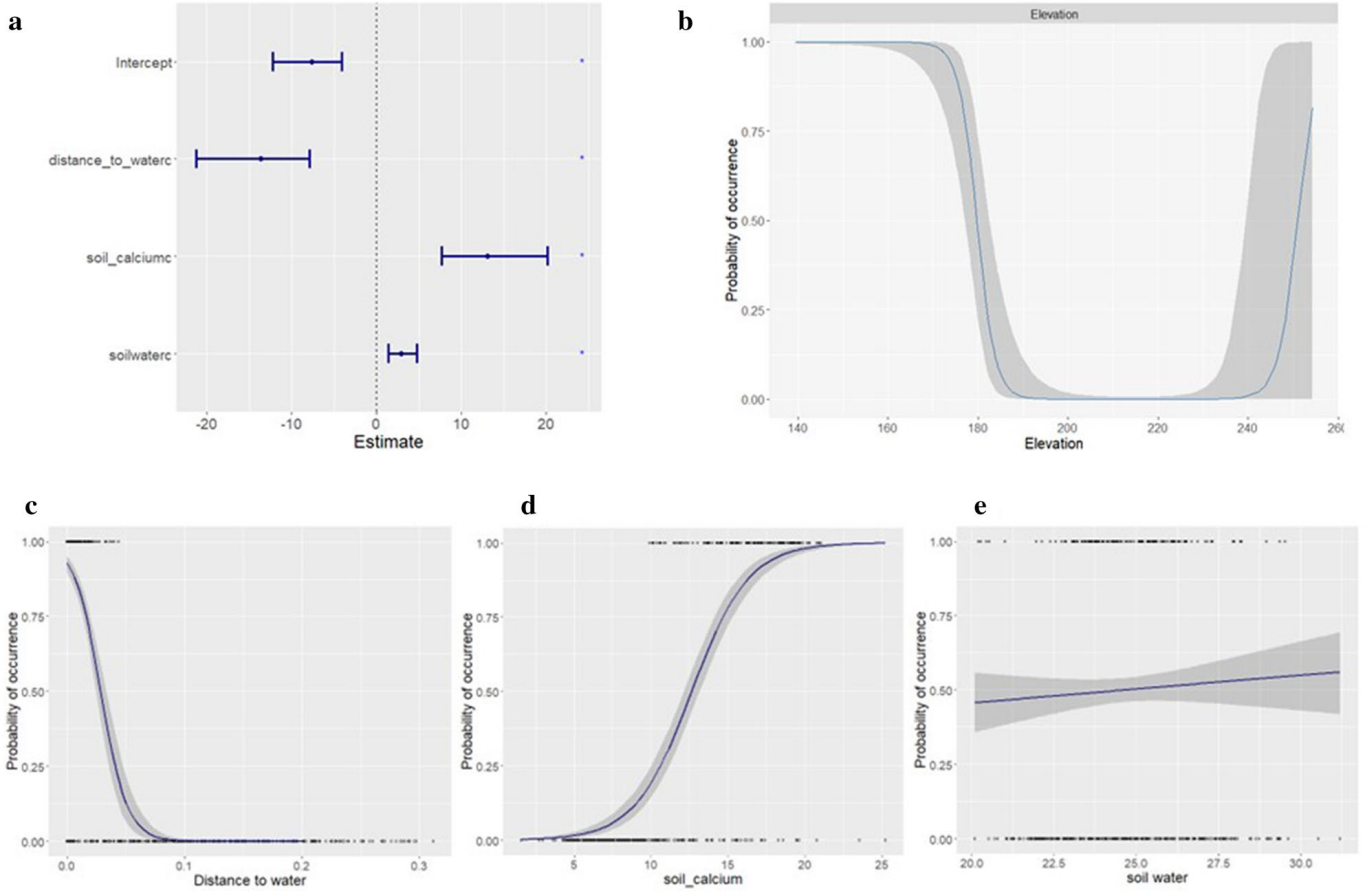
(**A**) The fixed effect and credible intervals for the linear covariates, (**B**) the smoothed fits of elevation (in m in x-axis) and linear fits of: (**C**) distance to water (in km in x-axis), (**D**) soil calcium (in cmolc/kg in x-axis), (**E**) soil water (in v% in x-axis) variables. The y-axis shows the estimated probability of presence. The shaded grey polygons represent 95% credible intervals.

The posterior predicted mean probability of *B. anthracis* suitability and the 95% credible intervals for the parameters of the fixed effects used in the final INLA model are shown in Fig. 3. The results demonstrated a negative relationship between distance to water and the presence of *B. anthracis* between 0 and 0.3 km. However, soil calcium had a positive association with the occurrence of *B. anthracis* between 10 and 25 cmolc/kg. Similarly, the results showed that higher occurrences of *B. anthracis* are expected in areas with soil water availability of between 20 and 30 v% (the volumetric soil water content defined as the volume of water for each unit volume of soil) and elevation of 140–190 m (Fig. 3). The omission rate of the final model against the withheld evaluation dataset was 10%, indicating a sensitivity of 90% (181 out of 202 = 89.6%; rounded up to 90%). Only 21 locations out of the 202 positive test locations were omitted (predicted to be unsuitable) by the model projection, however, these locations were all within 50 km of the nearest predicted high-risk area. The recent study of the environmental factors influencing the distribution of anthrax across Queen Elizabeth National Park in Uganda had 5.2% omission rate, correctly predicting 94.8% of the test points applied for model validation^33^. The other recent study of the distribution of *B. anthracis* across Africa had omission rates of 3.4%, 10%, and 5.9% when no thinning, 30 km thinning, and 50 km thinning was applied to the dataset^58^.

**Figure 4:**
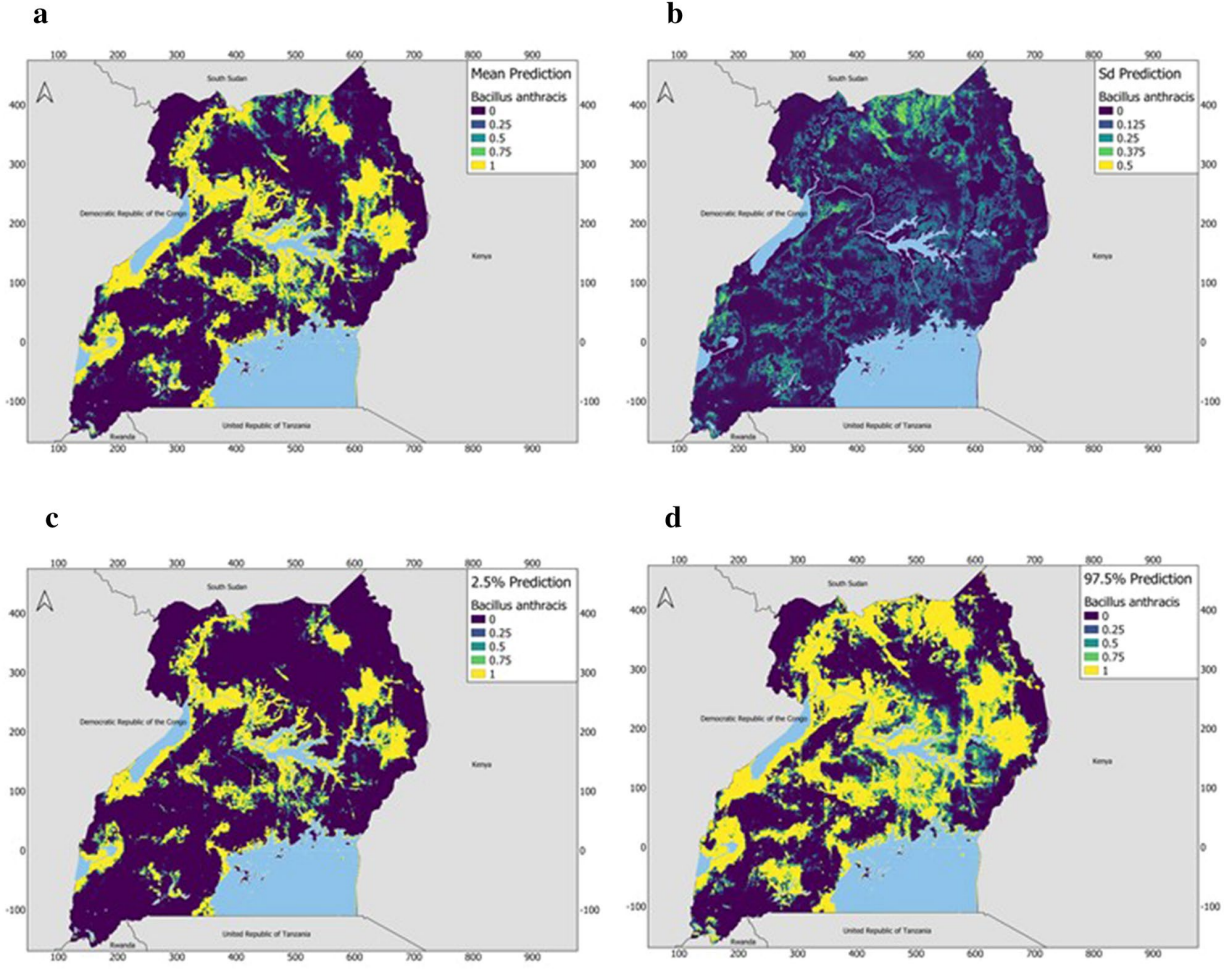
(**A**) The posterior predictive mean (**B**) posterior standard deviation (**C**) posterior lower credible interval (2.5% quantile) and (**D**) posterior credible upper interval (97.5% quantile) of the probability of *B. anthracis* suitability across Uganda from wildlife data (2004–2010). Prediction maps were developed using the Quantum Geographic Information System software (QGIS). URL: https://qgis.osgeo.org (2020).

**Figure 5:**
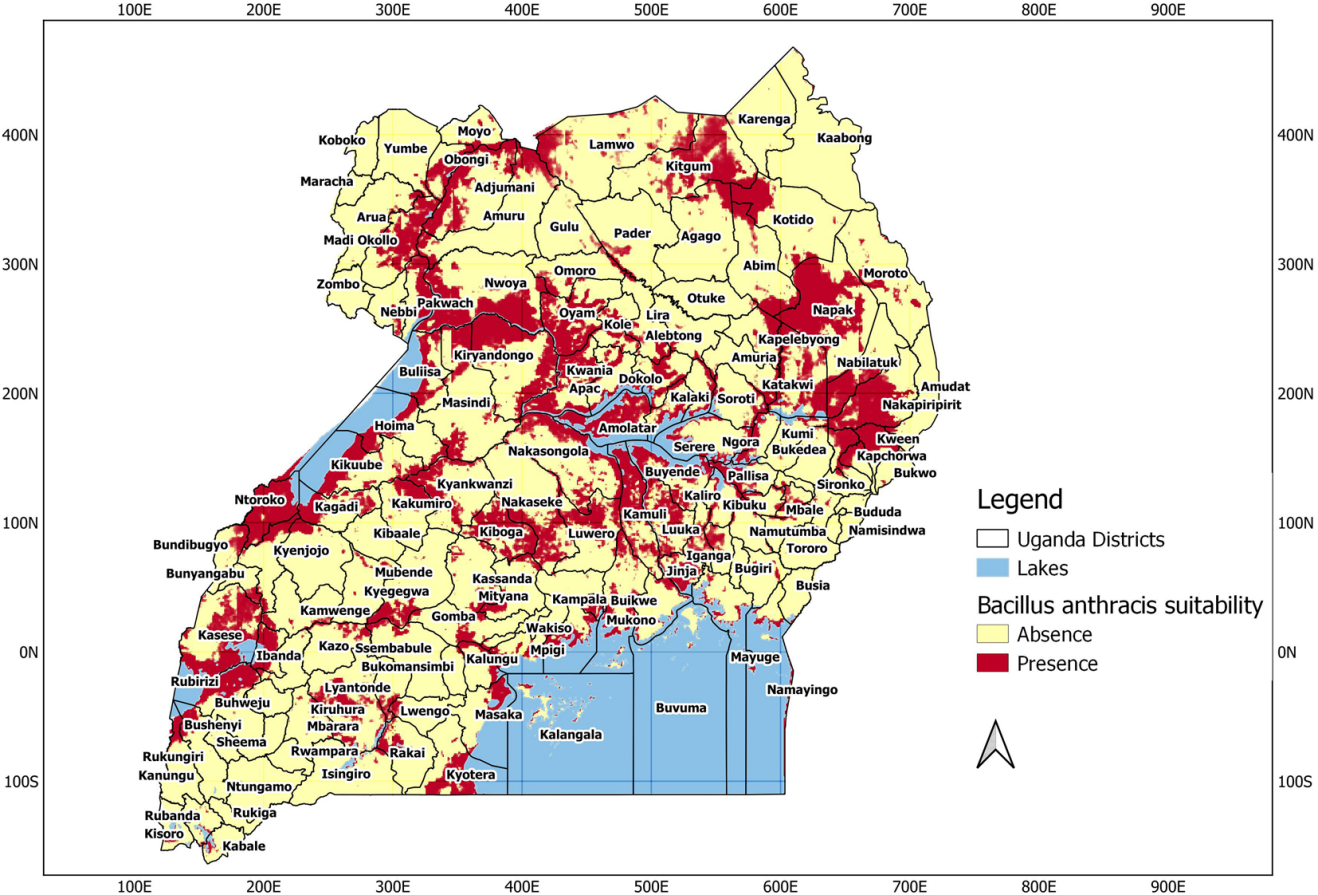
A binary map of the posterior predictive mean of the probability of *B. anthracis* suitability across Uganda using the threshold for maximum sensitivity and specificity (0.52). Prediction maps were developed using the Quantum Geographic Information System software (QGIS). URL: https://qgis.osgeo.org (2020).

The prediction maps from the INLA model identified several districts across Western, Central, Eastern, and Northern Uganda to be suitable for the occurrence of *B. anthracis* as shown in Table 3. These areas were identified to be regions that are likely to be suitable for the occurrence and persistent of *B. anthracis* (Fig. 4). The spatial effect which shows the intrinsic spatial variability of the observed data after excluding the environmental predictors, showed a low (almost negligible) effect and was, therefore, excluded from the final model, implying that the variability of the species data for *B. anthracis* appears to be explained by the chosen variables used in the final model. Figure 5 is a binary map created using the threshold for maximum sensitivity and specificity. It shows areas that are likely to be suitable for anthrax and those that are not.

**Table 3:** Districts across Uganda with moderate to high *B. anthracis* suitability by region.

Region	Districts
Western	Kasese, Rubirizi, Kitagwenda, Rukungiri, Kanungu, Kamwenge, Bunyangabu, Kabarole, Kazo, Kiruhura, Isingiro, Mbarara, Kabale, Kisoro, Kyegegwa. Bundibugyo, Kyenjojo, Ntoroko, Kagadi, Kakumiro, Kikuube, Hioma, Masindi, Buliisa, Kiryandongo
Central	Kyotera, Rakai, Lwengo, Lyantonde, Ssembabule, Mubende, Gomba, Kalungu, Kassanda, Mityana, Mpigi, Wakiso, Mukono, Kampala, Kayunga, Luwero, Nakaseke, Kiboga, Nakasongola, Kyankwanzi
Eastern	Kapchorwa, Kween, Bulambuli, Jinja, Kamuli, Buyende, Iganga, Luuka, Kaliro, Budaka, Kibuku, Pallisa, Ngora, Katakwi, Kapelebyong, Soroti, Serere, Kalaki, Kaberamaido
Northern	Kitgum, Kotido, Abim, Agago, Napak, Moroto, Nabilatuk, Nakapiripirit, Omoro, Oyam, Kole, Apak, Lira, Kwania, Dokolo, Abletong, Nwoya, Pakwach, Nebbi, Arua, Yumbe, Madi, Okollo, Obongi, Adjumani, Moyo, Amuru, and Lamwo

## Discussion

This study uses a Bayesian framework to model the suitability of *B. anthracis* using wildlife data from Queen Elizabeth National Park, as well as livestock and human data from the MAAIF and MOH in Uganda. We demonstrate the INLA Bayesian framework to be a method that can predict hotspots of disease-causing organisms and inform prevention and surveillance strategies. ENMs have gained rapid popularity as powerful disease epidemiology tools^23,24^. A major preliminary step to reducing anthrax outbreaks is to identify and prioritize areas that are suitable for the occurrence of *B. anthracis*^33^. The correct detection of such areas can enhance effective management strategies for anthrax prevention. However, this requires a detailed understanding of the spatial distribution of *B. anthracis*, given that misidentification of anthrax “hotspots” can cause erroneous outbreak prevention practices leading to considerable economic losses.

The Bayesian model results show that the suitability of *B. anthracis* is influenced by soil properties, topography, as well as proximity to permanent water bodies. The non-linear relationship between elevation and species distribution demonstrated by the models implies that *B. anthracis* may occur in areas with distinct environmental properties but displaying higher preferences for areas with lower elevation (around 140–190 m). The linear relationships suggest that *B. anthracis* may prefer areas closer to water bodies (between 0 and 0.3 km). These results concur with the findings from a recent study on *B. anthracis* suitability conducted in Kruger National Park in South Africa which showed that low altitude (between 225 and 280 m) and close proximity to a water bodies were essential for survival and persistence of anthrax^37^. Soil water availability had a weak positive effect on *B. anthracis* suitability. This variable, sometimes referred to as soil moisture has been shown to be an important predictor of *B. anthracis* suitability in Southern Kenya^36^.

High soil calcium concentrations (10–25 cmolc/kg) also had a significant positive effect on the suitability of and area to *B. anthracis*. A recent study of *B. anthracis* suitability in Queen Elizabeth National Park in Uganda reported exchangeable calcium to be an important contributor in the creation of a species survival model for the bacteria^33^. Steenkamp et al.^37^ also reported that high calcium concentration was a significant driver of anthrax occurrence in South Africa. A growing body of evidence supports the fact that calcium has an important role in the preservation of anthrax spores in the soil^59^. Studies have shown that calcium can be absorbed from the environment by the bacteria during spore formation and incorporated into the layers of the bacterial spore, particularly the core^60,61^. Calcium then combines with diplicolinic acid to form a salt lattice that helps to stabilize the enzymes and DNA in the spore, hence, helping the bacteria to maintain dormancy and develop thermoresistance properties^60–62^.

The INLA framework also allows the use of Delaunay triangulation as opposed to the regular grid structures that are commonly applied in conventional ENMs. This approach gathers more information from the areas with more observed data; thus, the density of triangulation is higher across these regions contributing to more accurate predictions. This approach considers the boundary effect by creating a mesh that transitions smoothly from regions dominated by the smaller triangles (domain of interest in the model) to the regions with the large triangles (areas outside the domain of interest that help to avoid boundary effects within the model).

The areas of Western Uganda within Queen Elizabeth National Park, Northern Uganda around Arua, and Kween district in Eastern Uganda were predicted to be highly suitable for *B. anthracis* occurrence, confirming the results of previous studies with the accurate detection of the most suitable areas for the occurrence of the species^33,58^. Compared to the most recent attempt to model the distribution of anthrax in Uganda^33^, our model was different with regards to the parameters included and some of the covariates that were selected. For instance, the inclusion of the spatial random effect during model selection allowed the consideration of the effect of spatial autocorrelation before and after the addition of the covariates. The spatial effect was eventually excluded from the final model because the covariates used were able to account for most of the variation observed within the observed data. However, it is still important for future studies to test for any residual spatial variation and, if present, account for this and INLA provides a computationally efficient way of achieving this.

The INLA Bayesian framework applies probability distributions for the posterior estimates to model uncertainty within the parameter values^23^. As such, a point estimate of the posterior probability of suitability as well as the uncertainty surrounding it can be obtained and assessed^24^. Estimating this uncertainty via spatial maps is vital to offering end-users a realistic species suitability distribution to identify prevention and surveillance options. INLA models can not only deal with smoothing techniques such as GAMs, but they can also conduct inference and model prediction simultaneously, handle missing data, and incorporate biases within the data as spatial random effects, e.g., survey effort. One key limitation of this study is the lack of systematically collected and detailed anthrax case information. Therefore, it was impossible to account for structural dependencies such as temporal autocorrelation and analytical biases like survey effort or surveillance efficiency. Future studies should try to consider the incorporation of survey effort as well as temporal autocorrelation as model offsets or as other explanatory covariates within the model particularly when modelling incidence.

This work employs a Bayesian GAM via INLA to examine the spatial suitability of *B. anthracis* using data from MAAIF, MOH, and published studies in Uganda. R-INLA has been shown to generate model predictions of species occurrence, which are better than those generated by Maxent and Boosted Regression Trees especially when the occurrence dataset is clumped or restricted^18^. Our model was able to pick up known locations of anthrax outbreaks occurring 8 years after the dataset used to calibrate the model with a sensitivity of 90%. The 10% of the test locations that were omitted by the model projection were all within 50 km of the nearest predicted high-risk area. There is a possibility that the animals could have acquired the infection within a high-risk area and died at a different location which was then georeferenced. These results demonstrate a high level of precision, making the prediction maps useful enough to be incorporated into future anthrax prevention and surveillance plans by the relevant stakeholders in Uganda. The model used in the past study to map the distribution of anthrax across Queen Elizabeth National Park in Uganda had a lower omission rate (5.2%) and slightly higher sensitivity of 94.8% against the test points^33^. However, the occurrence data points used in the study were split randomly by a ratio of 3:1, with 75% of the data applied in model calibration, and the remaining 25% in model validation^33^. The other recent continental study of the distribution of *B. anthracis* across Africa also had lower omission rates when no thinning (3.4%) and 50 km thinning (5.9%) were applied to the datasets^58^. However, like the Queen Elizabeth study, the models were created by randomly sampling 50% of the occurrence dataset for model calibration and the rest (50%) for model validation^58^. Random partitioning of the data into training and testing sets can inflate the performance of a model and underestimate the error in the spatial prediction evaluation^50^.

The overall achievement of this study was to generate novel and highly relevant predictions of the spatial distribution of *B. anthracis* suitability across Uganda. Anthrax emerged as the top disease of highest priority for human and animal health sectors during the Ugandan One Health Zoonotic Disease Prioritization Workshop in 2017^24^. The key recommendations from this workshop included an emphasis on building laboratory capacity, enhancing surveillance, improving outbreak response, and focusing on prevention and control, particularly using a multi-sectoral, One Health approach. The suitability maps we have generated in this study will help policymakers in Uganda to effectively estimate the cost of implementing targeted anthrax prevention and surveillance campaigns and plan their management decisions effectively. This study also provides a basis for targeted studies to validate and improve predictions.

## Conclusion

This work used a Bayesian framework to model the suitability of *B. anthracis* using data from MAAIF, MOH, and published studies in Uganda. The model identified known locations of anthrax outbreaks in Kiruhura, Arua, Pakwach, and Kween Districts, occurring 8 years later with excellent sensitivity. The prediction maps generated here can guide future anthrax prevention and surveillance plans by relevant stakeholders in Uganda. INLA methods can account for the analytical biases and structural dependencies within the input data, but more effort still needs to be put in place to allow for detailed and systematic data surveillance to improve the quality of observation data available.

## Data Availability

The datasets and R code supporting the conclusions of this article are available in the GitHub repository, https://github.com/spatialmodels/Uganda-Spatial_Model.
